# Identification of Naturally Processed Hepatitis C Virus-Derived Major Histocompatibility Complex Class I Ligands

**DOI:** 10.1371/journal.pone.0029286

**Published:** 2012-01-03

**Authors:** Benno Wölk, Claudia Trautwein, Benjamin Büchele, Nadine Kersting, Hubert E. Blum, Hans-Georg Rammensee, Andreas Cerny, Stefan Stevanovic, Darius Moradpour, Volker Brass

**Affiliations:** 1 Department of Medicine II, University of Freiburg, Freiburg, Germany; 2 Institute of Virology, Hannover Medical School, Hannover, Germany; 3 Department of Immunology, University of Tübingen, Tübingen, Germany; 4 Clinical Pharmacology and Clinical Immunology/Allergology, Inselspital, University of Bern, Bern, Switzerland; 5 Division of Gastroenterology and Hepatology, Centre Hospitalier Universitaire Vaudois, University of Lausanne, Lausanne, Switzerland; Singapore Institute for Clinical Sciences, Singapore

## Abstract

Fine mapping of human cytotoxic T lymphocyte (CTL) responses against hepatitis C virus (HCV) is based on external loading of target cells with synthetic peptides which are either derived from prediction algorithms or from overlapping peptide libraries. These strategies do not address putative host and viral mechanisms which may alter processing as well as presentation of CTL epitopes. Therefore, the aim of this proof-of-concept study was to identify naturally processed HCV-derived major histocompatibility complex (MHC) class I ligands. To this end, continuous human cell lines were engineered to inducibly express HCV proteins and to constitutively express high levels of functional HLA-A2. These cell lines were recognized in an HLA-A2-restricted manner by HCV-specific CTLs. Ligands eluted from HLA-A2 molecules isolated from large-scale cultures of these cell lines were separated by high performance liquid chromatography and further analyzed by electrospray ionization quadrupole time of flight mass spectrometry (MS)/tandem MS. These analyses allowed the identification of two HLA-A2-restricted epitopes derived from HCV nonstructural proteins (NS) 3 and 5B (NS3_1406–1415_ and NS5B_2594–2602_). In conclusion, we describe a general strategy that may be useful to investigate HCV pathogenesis and may contribute to the development of preventive and therapeutic vaccines in the future.

## Introduction

With an estimated 120–180 million chronically infected individuals, HCV is a leading cause of chronic hepatitis, liver cirrhosis and hepatocellular carcinoma worldwide [1]. Antiviral therapy has improved considerably with the introduction of pegylated interferon-α and ribavirin as well as, more recently, the first generation of directly acting antivirals. However, many patients still do not respond to or cannot tolerate antiviral therapy. In addition, HCV continues to be transmitted in certain areas of the world [2]. Therefore, the development of preventive and therapeutic vaccines against hepatitis C is of major public health importance [3].

Innate and adaptive immune responses to HCV have been studied in great detail [4]. Resolution of acute hepatitis C correlates with the induction of strong and broad CD4+ and CD8+ T cell responses [5]. However, the majority of patients fail to eliminate HCV and develop chronic infection (reviewed in [5], [6]). The high genetic variability of HCV significantly contributes to the escape from the immune system and complicates the development of an efficient vaccine [7]. Nevertheless, more recent data indicate that there is protective immunity against HCV [4].

A critical step for the understanding of the immunopathogenesis of HCV infection and HCV clearance is the presentation of viral epitopes on MHC class I molecules from infected cells. Most of the currently available experimental systems are limited, since an *a priori* defined set of synthetic peptides is used to either externally load target cells or to expand epitope-specific CD8+ T cells which are then used in downstream readout applications.

Therefore, the aim of this study was to identify specific MHC class I ligands which are naturally processed and presented by cells expressing HCV proteins. To this end, we engineered continuous human cell lines to inducibly express HCV proteins and to constitutively express high levels of functional HLA-A2. MHC class I molecules were isolated from large-scale cultures of these cell lines, followed by elution and identification of naturally processed CTL epitopes. This proof-of-concept study allowed the identification of two naturally processed HCV-derived MHC class I ligands. Although both epitopes have been described previously by conventional T-cell dependent methods, this novel approach has the potential to identify novel and unconventional epitopes.

## Results

### Generation of stable cell lines inducibly expressing HCV proteins and constitutively expressing functional HLA-A2

**Figure 1:**
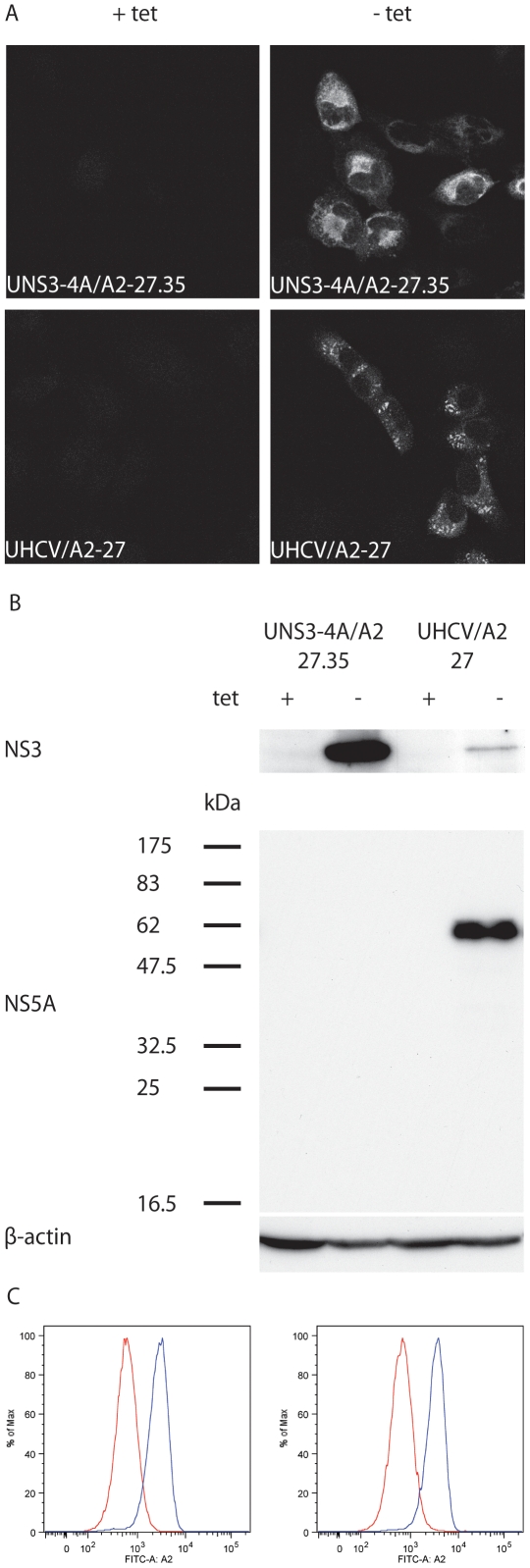
Cell lines UNS3-4A/A2-27.35 and UHCV/A2-27. (A) Indirect immunofluorescence microscopy of UNS3-4A/A2-27.35 and UHCV/A2-27 cells cultured for 48 h in the presence (+ tet) or absence (− tet) of tetracycline. Monoclonal antibody 1B6 against HCV NS3 was used as primary antibody. (B) Immunoblot analysis of UNS3-4A/A2-27.35 and UHCV/A2-27 cells were cultured for 48 h in the presence (+ tet) or absence (− tet) of tetracycline. Monoclonal antibodies 1B6 against HCV NS3 or 11H against NS5A were used as primary antibodies. (C) Left panel: HLA-A2 surface expression of UNS3-4A/A2-27.35 (blue histogram) compared to the founder cell line UNS3-4A-24 (red histogram). Right panel: HLA-A2 surface expression of UHCV/A2-27 (blue histogram) compared to the founder cell line UHCVcon-57.3 (red histogram).

To identify naturally processed HCV MHC class I ligands by mass spectrometry (MS), as described in this manuscript, U-2 OS human osteosarcoma-derived cell lines UNS3-4A-24 and UHCVcon-57.3 [8], [9] were used. These cells allow for tight, tetracycline-regulated expression of the HCV nonstructural protein 3-4A (NS3-4A) complex or of the entire viral polyprotein, respectively. However, U-2 OS cells express low levels of endogenous HLA-A2 [10]. To enhance MHC class I presentation of HCV antigens, UNS3-4A-24 and UHCVcon-57.3 cells were engineered to overexpress HLA-A2. Clones UNS3-4A/A2-27.35 and UHCV/A2-27 were used in this study. As shown in Figure 1 (panels A and B), these cells allow for tightly regulated expression of HCV proteins, as documented by immunoblot and indirect immunofluorescence microscopy. NS3 was identified in both cell lines at the expected molecular mass of 70 kDa. Detection of NS5A at the expected molecular mass of 56 kDa demonstrates correct viral polyprotein processing in UHCV/A2-27 cells. Furthermore, cells were analyzed for HLA-A2 surface expression by flow cytometry (Fig. 1C). Compared to the founder cells, both cell lines showed an enhanced level of HLA-A2 surface expression. Furthermore, strong HLA-A2 expression could also be detected after withdrawal of tetracycline from the culture medium and induction of HCV protein expression (data not shown). This is a prerequisite for the elution of HCV proteins from MHC class I molecules isolated from these cells and demonstrates that HCV protein expression does not interfere with HLA-A2 surface expression.

### Targeting of UNS3-4A/A2-27.35 and UHCV/A2-27 cells by HCV-specific CD8+ CTL

**Figure 2:**
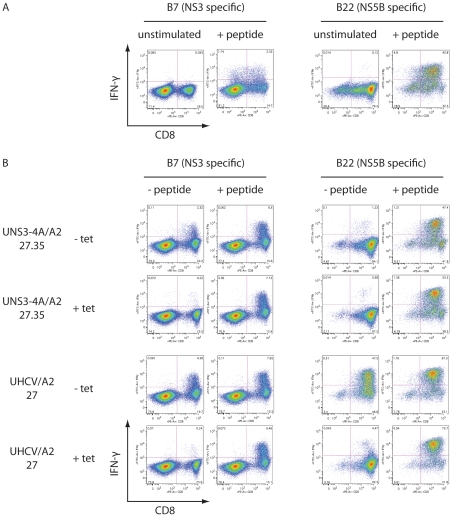
Targeting of UNS3-4A/A2-27.35 and UHCV/A2-27 cells by HCV-specific CD8+ CTL. CD8+ T cell lines B7 and B22, targeting epitopes CINGVCWTV derived from NS3 and ALYDVVTKL localized in NS5B, respectively, were used for coculture experiments. (**A**) Stimulation of T cell lines with the corresponding synthetic peptide leads to their activation, as indicated by the expression of IFN-γ. For all dot blots in this figure, CD8 is displayed on the abscissa and IFN-γ expression on the ordinate. (**B**) UNS3-4A/A2-27.35 and UHCV/A2-27 cells cultured for 48 h in the presence (+ tet) or absence (− tet) of tetracycline were cocultured with CD8+ T cell lines. In the presence of tetracycline, no substantial activation of the T cell lines could be observed. However, strong activation was observed after loading of UNS3-4A/A2-27.35 and UHCV/A2-27 cells with the corresponding synthetic peptide. By contrast, the NS3-specific B7 cell line was activated efficiently by UNS3-4A/A2-27.35 and UHCV/A2-27 cells cultured in the absence of tetracycline while the NS5B-specific B22 cell line could be stimulated only by UHCV/A2-27 cells, which upon tetracycline withdrawal express the entire HCV polyprotein. When target cells were cultured in the absence of tetracycline, T cell activation was enhanced only slightly by external peptide loading.

To explore whether UNS3-4A/A2-27.35 and UHCV/A2-27 cells can serve as targets for HCV-specific CTL, cell lines were cocultured with CD8+ T cell lines that have been raised to recognize epitopes localized in NS3 (B7, CINGVCWTV) or NS5B (B22, ALYDVVTKL), respectively. T cell activation was assessed by intracellular IFN-γ staining and flow cytometry. As shown in Figure 2A, CTL lines were activated specifically after external loading with the corresponding synthetic peptide. More important, coculture of UNS3-4A/A2-27.35 and UHCV/A2-27 cells with CD8+ T cell lines demonstrated their potential to endogenously express and functionally present HCV epitopes (Fig. 2B). Coculture of UNS3-4A/A2-27.35 and UHCV/A2-27 cells leads to efficient activation of the CD8+ T cell line which recognizes the B7 epitope located in NS3. By contrast, and as expected, the CD8+ T cell line specific for the B22 epitope in NS5B recognizes only UHCV/A2-27 cells. No CTL reactivity was observed when target cells were cultured in the presence of tetracycline, i.e. when they did not express HCV proteins. These results validate both cell lines as functional targets for HCV-specific CD8+ T cells and demonstrate that naturally processed HCV epitopes are efficiently presented on HLA-A2 molecules in this model system.

### Large-scale expansion of UNS3-4A/A2-27.35 and UHCV/A2-27 cells, isolation of MHC class I molecules and peptide elution

**Figure 3:**
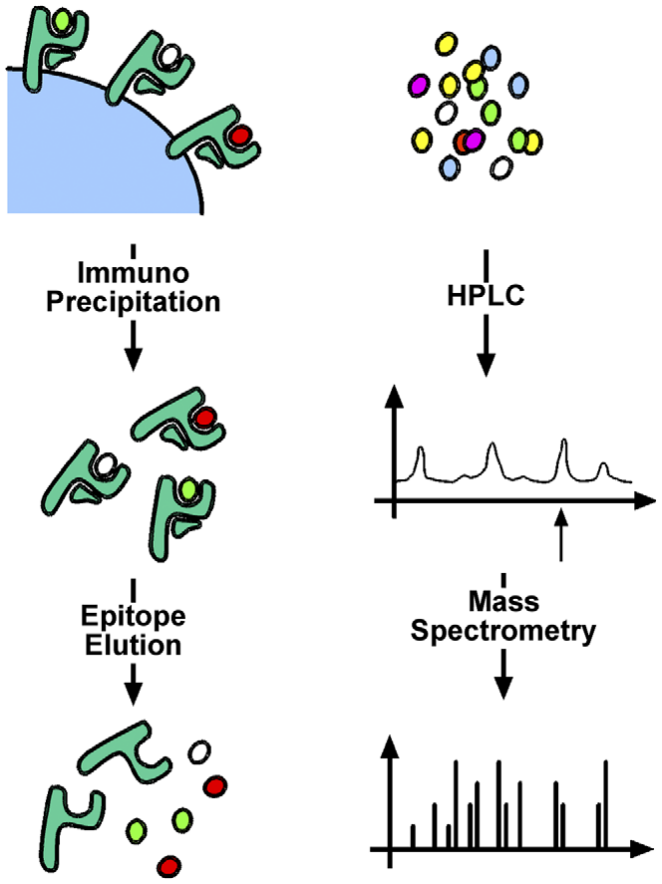
Identification of naturally processed HLA-A2 ligands. Following large-scale expansion, cell pellets were lysed and MHC class I molecules with bound ligands were purified by immunoprecipitation. Subsequently, ligands were eluted and separated by high performance liquid chromatography (HPLC). Fractions of interest were sequenced by mass spectrometry.

To obtain the required amounts of HLA-A2 with naturally processed ligands, UNS3-4A/A2-27.35 and UHCV/A2-27 cells were expanded to large scale, yielding cell pellets of 60 ml each. From these, 1.5 and 7.0 nmol MHC class I molecules were isolated, respectively. The yield of HLA molecules (as determined by Edman degradation) corresponds to 25 and 117 pmol per gram of cells, respectively. According to our experience, such values are typical for tumor cells that do not have abundant HLA expression [11]. The higher yield from UHCV/A2-27 cells presumably correlates with higher HLA-A2 expression. Subsequently, ligands were eluted from MHC class I molecules, as described in the Materials and Methods section and as illustrated schematically in Figure 3.

### Detection of HCV-derived peptides in MHC class I ligands isolated from UNS3-4A/A2-27.35 and UHCV/A2-27 cells

**Figure 4:**
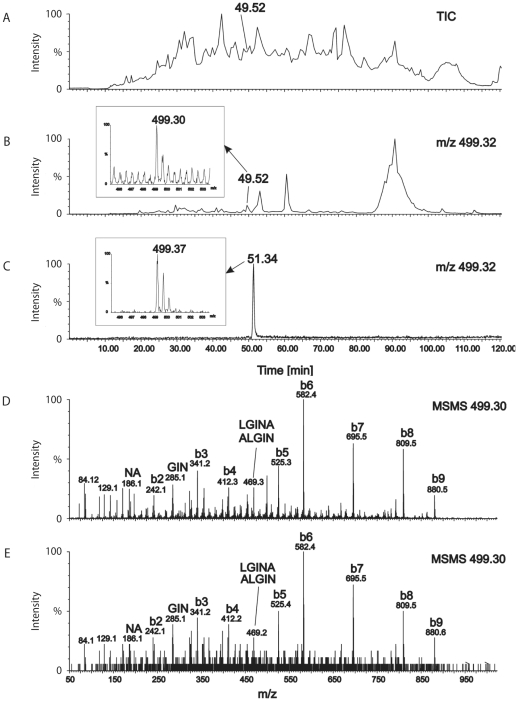
Detection and characterization of peptide NS3_1406–1415_ in MHC class I ligands isolated from UNS3-4A/A2-27.35 cells. Naturally processed MHC class I ligands of UNS3-4A/A2-27.35 cells were separated by high performance liquid chromatography (HPLC) and analyzed by mass spectrometry. (A) A chromatogram of the total ion current (TIC) is shown. (B) The mass chromatogram of ionized peptides with m/z = 499.3 reveals a peak at retention time t = 49.5 min that correlates with (C) a signal of the synthetic peptide NS3_1406–1415_ (KLVALGINAV) which elutes after 51.3 min. In all graphs, HPLC retention times are shown on the abscissa and relative signal intensity on the ordinate. Small insets in panels B and C show mass spectra of peptides eluted at the indicated time points. (D) MSMS spectrum of the synthetic peptide NS3_1406–1415_ (KLVALGINAV). (E) MSMS spectrum of the natural peptide isolated from UNS3-4A/A2-27.35 cells, revealing amino acid sequence KLVALGINAV identical to the synthetic peptide shown in panel A. Identified amino acid sequences of peptide fragments are indicated on the top of the peaks. Due to the protonated N-terminal lysine residue, the b series of peptide fragments is dominating the MSMS spectra.

Naturally processed MHC class I ligands of UNS3-4A/A2-27.35 and UHCV/A2-27 cells were separated by high performance liquid chromatography (HPLC) and analyzed by electrospray ionization (ESI) quadrupole time of flight (Q-TOF) mass spectrometry (MS)/tandem MS, as described in the Materials and Methods section. HPLC and MS of predicted synthetic peptides allowed to screen for epitopes of interest in ligand preparations and subsequent sequence verification by online liquid chromatography (LC) MS analysis. With this approach, two naturally processed MHC class I ligands could be identified. The peptide isolated from UNS3-4A/A2-27.35 cells is derived from NS3 (HCV polyprotein position 1406–1415; NS3-4A_1406–1415_) with the amino acid sequence KLVALGINAV (Fig. 4). The epitope isolated from UHCV/A2-27 cells is derived from NS5B (HCV polyprotein position 2594–2602; NS5B_2594–2602_) with the amino acid sequence ALYDVVSKL (Figures S1 and S2) that is cross-recognized by epitope ALYDVVTKL-specific T cells (Fig. 2) in spite of the threonine instead of serine in position 2600.

## Discussion

In this proof-of-concept study, we identified two naturally processed HCV-derived MHC class I ligands. These were isolated from cell lines UNS3-4A/A2-27.35 and UHCV/A2-27, allowing the tightly regulated expression of HCV NS3-4A and of the entire viral polyprotein, respectively, as well as the constitutive expression of functional HLA-A2. These cell lines were specifically recognized by HCV-specific CTL, indicating efficient presentation of naturally processed MHC class I ligands. MHC class I molecules were purified by immunoprecipitation. Ligands were eluted, separated by HPLC and sequenced by ESI Q-TOF-MS/MS. Among other MHC class I ligands, two naturally processed HCV epitopes, NS3_1406–1415_ and NS5B_2594–2602_ were identified. Interestingly, both had been described previously, based on prediction algorithms [12], [13].

HCV replication occurs within the cytoplasm of infected cells. Translation and processing of the viral polyprotein take place in close association with membranes of the endoplasmic reticulum. HCV proteins are then subjected to cellular antigen-processing pathways, resulting in the presentation of HCV epitopes on the cell surface. It has been estimated that approx. 2,000–10,000 molecules of a protein are required to allow the presentation of one antigen [14]. In fact, antigen processing involves a highly complex interplay of multiple steps and factors. Degradation by the proteasome, interaction with chaperones such as calnexin, incorporation into the peptide loading complex, involving other chaperones such as tapasin, peptide trimming by aminopeptidases, loading onto empty MHC I molecules, and, finally, transport across the secretory pathway to the cell surface are pivotal steps that are tightly coordinated during this process. Several approaches typically used to identify MHC I epitopes bypass this complex process, which could be one reason why they eventually fail to identify relevant epitopes. These approaches include (i) the induction and identification of T cells through the stimulation with defined synthetic peptides and (ii) the generation of epitope-specific T cell lines which are subsequently tested for their reactivity with cells expressing a given antigen. There are several potential limitations of these systems as they are restricted by the use of synthetic peptides or depend on the presence of functional HCV-specific T cells.

Against this background, a direct T cell-independent identification of naturally processed MHC class I epitopes would be desirable. In this context, we have demonstrated that peptide elution from HCV protein-expressing cell lines and subsequent direct sequencing by ESI Q-TOF MS/MS is a feasible and powerful approach. Using this experimental setting, UNS3-4A/A2-27.35 and UHCV/A2-27 cells turned out to be valuable tools which could be further employed to study HCV antigen processing and presentation in the future. The identification of two known HCV HLA-A2 ligands that are localized in NS3 and NS5B demonstrates their authentic processing and presentation *in vivo*. Furthermore, a more elaborate approach, using tracer substances such as isotope-labeled peptides, might hold promise for the quantification of an epitope relative to the complete repertoire of presented ligands. Few reports on Epstein-Barr virus-encoded proteins investigated how the amount of presented MHC I antigen complex could influence the efficiency of recognition by CD8+ T cells [15], [16]. Therefore, in addition to the proof-of-principle of this particular experimental setting, the identification of the two epitopes by this novel approach underlines their importance as natural targets for HCV-specific T cells. CTL responses against these epitopes could be of particular importance to control viral infection and may be included as targets in future vaccination strategies.

In principle, the direct sequencing of MHC I ligands should allow to identify epitopes after posttranslational protein modifications such as glycosylation, phosphorylation and proteolytic processing as well as unconventional epitopes derived from alternative reading frames or RNA splicing that are not detected by the current conventional methods. Future studies aimed at identifying naturally processed HCV-derived MHC class I ligands may provide novel insights into epitope processing and presentation as well as recognition, thereby contributing to the understanding of HCV pathogenesis.

## Materials and Methods

### Establishment and characterization of inducible cell lines

U-2 OS human osteosarcoma-derived cell lines inducibly expressing the HCV NS3-4A complex (UNS3-4A-24) or the entire HCV polyprotein (UHCVcon-57.3) derived from the HCV H77 isolate (genotype 1a) have been described previously [8], [9]. These cells were stably transfected with a cytomegalovirus (CMV) promoter-driven genomic HLA-A2 construct to augment MHC class I expression, as described previously [10]. In brief, cells were cotransfected with pCMV/HLA-A2 and pTK-Hyg (Clontech, Palo Alto, CA) followed by selection with 500 µg/ml G418 (for the selection of the tetracycline-regulated transactivator, tTA), 1 µg/ml puromycin (for the selection of the HCV expression construct driven by a tTA-dependent promoter), and 100 µg/ml hygromycin (for the selection of the HLA-A2 expression construct). Stable transfectants were cloned and screened for high-level HLA-A2 expression by flow cytometry, as specified below. Tightly regulated expression of HCV proteins was confirmed by immunoblot analyses which were performed as described previously [17]. Clones UNS3-4A/A2-27.35 and UHCV/A2-27 were selected for further experiments.

### Immunofluorescence microscopy

Indirect Immunofluorescence was performed as described previously [17]. The specific antibodies against NS3 (1B6) and NS5A (11H) have been described elsewhere [8], [18].

### Peptides and antibodies for flow cytometry

Peptides with the amino acid sequence CINGVCWTV and ALYDVVTKL were synthesized with free amino and carboxyl termini by standard Fmoc chemistry (Genaxxon BioScience, Ulm, Germany). The peptides were dissolved and diluted as described previously [19]. Fluorescein isothiocyanate- or phycoerythrin-labeled anti-HLA-A2, anti-CD8 and anti-IFN-γ antibodies as well as isotype-matched control antibodies were from Becton Dickinson (Heidelberg, Germany) and were used following the manufacturer's recommendations.

### CD8+ T cell lines

Blood samples were obtained from two HLA-A2-positive patients with chronic HCV infection after written informed consent and in accordance with the 1975 Declaration of Helsinki, federal guidelines as well as the local ethics committee as described previously [20]. T cell lines raised against the epitopes CINGVCWTV and ALYDVVTKL, localized within NS3 and NS5B, respectively, were referred to as B7 and B22.

### Intracellular IFN-γ staining

Intracellular IFN-γ staining was performed essentially as described [19]. In brief, T cells (2×10^5^ per 96-well) were stimulated either directly with peptides (10 µg/ml) or by UNS3-4A/A2-27.35 and UHCV/A2-27 cell lines in the presence of 50 U/ml human recombinant interleukin 2 (Hoffmann La Roche, Basel, Switzerland) and 1 µl/ml brefeldin A (Becton Dickinson, Heidelberg, Germany). As a positive control, UNS3-4A/A2-27.35 and UHCV/A2-27 were externally loaded with the corresponding synthetic peptide before the coculture. Cocultivation experiments of T cell lines with UNS3-4A/A2-27.35 and UHCV/A2-27 cells were performed at an effector-to-target ratio of 1∶1. After 5 h of incubation (37°C, 5% CO_2_), cells from each well were blocked with IgG_1_ antibodies and stained with antibodies against CD8. After permeabilization with Cytofix/Cytoperm (Becton Dickinson, Heidelberg, Germany), cells were stained with antibodies against IFN-γ and fixed in 2% paraformaldehyde. Samples were acquired on a FACS Canto II flow cytometer (Becton Dickinson, Heidelberg, Germany) and analyzed with FlowJo v8.8.6 software (TreeStar, Ashland, OR).

### Cell culture and large-scale expansion

UNS3-4A/A2-27.35 and UHCV/A2-27 cells were grown in Dulbecco's modified Eagle medium (DMEM), 10% fetal bovine serum, 500 µg/ml G418, 1 µg/ml puromycin, 100 µg/ml hygromycin and 1 µg/ml tetracycline. For large-scale expansion, cells were initially expanded in 15-cm culture dishes and then seeded into a forty-tray array (Nunc Cell Factories, Nalge Nunc International, Naperville, IL). Tetracycline was withdrawn to induce expression of the HCV proteins, followed one week later by detachment with Accutase (PAA, Pasching, Austria), harvesting and washing of the cell pellets.

### Isolation of MHC class I molecules, peptide elution and sequencing

HLA-presented peptides were obtained by immunoprecipitation of HLA molecules from cell lines UNS3-4A/A2-27.35 and UHCV/A2-27. One volume of 2× lysis buffer containing PBS, 0.6% CHAPS, and complete protease inhibitor (Roche Diagnostics, Basel, Switzerland) was added to shock-frozen cell pellets. Subsequently, cells were homogenized by stirring for 1 h at 4°C. After four times 30 s sonication, the sample was stirred again for 1 h and subsequently centrifuged at 3,000× *g*, 4°C for 20 min, and afterwards at 150,000× *g*, 4°C for 1 h, to remove cell debris. Finally, the supernatant was passed through a 0.2-µm filter (Sartorius, Göttingen, Germany).

For immunoprecipitation, lysates were applied for at least 12 h to a CNBr-activated Sepharose 4B column (40 mg Sepharose per mg tissue; GE Healthcare, München, Germany) to which the HLA-A-, HLA-B-, and HLA-C-specific antibody W6/32 had been coupled (1 mg antibody per mg tissue), as described by the manufacturer. After binding, the column was rinsed with 250 ml PBS and subsequently with 500 ml double-distilled water. For elution and dissociation of bound HLA-peptide complexes, the column was shaken at least four times in one bed volume of 0.1% trifluoroacetic acid (TFA) for 20 min at room temperature. The four TFA eluates were subsequently collected and combined. Finally, the HLA-presented peptides were isolated by ultrafiltration through a Centricon 10-kDa cutoff membrane (Millipore, Billerica, MA). For LC-MS, samples were freeze-dried and resuspended in 0.1% formic acid.

HLA-extracted peptide pools were separated by reverse-phase HPLC (Ultimate Dionex, Amsterdam, Netherlands) and analyzed by nano-ESI MS on a Q-TOF MS/tandem MS (Micromass, Manchester, UK), as described [21]. Fragment spectra were analyzed manually and database searches (National Center for Biotechnology Information) were carried out using Multiple Alignment System for Protein Sequences Based on Three-way Dynamic Programming (MASCOT, http://www.matrixscience.com). Mass chromatograms of ionized synthetic peptides NS3_1406–1415_ (KLVALGINAV) and NS5B_2594–2602_ (ALYDVVSKL) were used as references to identify peaks of interest.

## Supporting Information

Figure S1 **Detection of peptide NS5B_2594–2602_ in MHC class I ligands isolated from UHCV/A2-27 cells.** Naturally processed MHC class I ligands of UHCV/A2-27 cells were separated by high performance liquid chromatography (HPLC) and analyzed by mass spectrometry. (A) Mass chromatogram of the total ion current (TIC). (B) In the mass chromatogram for m/z = 504.3 a signal peak is detected at retention time t = 63.5 min which was further analyzed by tandem mass spectrometry (MS/MS) (cf. Fig. S2).
(TIF)

Figure S2 **Identification of the NS5B_2594–2602_ epitope in UHCV/A2-27 cell-derived MHC class I ligands.** (A) UHCV/A2-27 cell-derived peptide (cf. Fig. S1) and (B) the synthetic peptide NS5B_2594–2602_ (ALYDVVSKL) were both detected with m/z = 504.3 at a retention time of t = 63.2 min and subsequently analyzed by tandem mass spectrometry (MS/MS). Fragmentation spectra of the synthetic peptide and the natural peptide are shown in panels A and B, respectively. Analysis of the natural peptide reveals amino acid sequence ALYDVVSKL and is identical to that of the synthetic peptide. Identified amino acid sequences of peptide fragments are indicated on top of the peaks. Due to the protonated lysine residue close to the C-terminal residue, the y series of peptide fragments is dominating the MSMS spectra.
(TIF)
